# The role of cardiac MR in patients successfully resuscitated from out of hospital sudden cardiac arrest: 5 year experience from a UK tertiary referral cardiac imaging centre

**DOI:** 10.1186/1532-429X-16-S1-P230

**Published:** 2014-01-16

**Authors:** Ausami Abbas, Faraz T Sheikh, Geraint Morton, James Shambrook, Charles Peebles

**Affiliations:** 1 Cardiothoracic Radiology, University Hospital Southampton, Southampton, UK; 2 Cardiology, University Hospital Southampton, Southampton, UK

## Background

Sudden cardiac arrest has a significant global health burden with an estimated incidence of 95.9 per 100 000 person-years. The causal malignant ventricular arrhythmia may arise secondary to myocardial ischaemia, infarct, oedema, fibrosis or infiltration and survivors are at significant risk for recurrent events. Coronary revascularization procedures have a significant associated morbidity/mortaility in this patient popuation and hence identifying those patients who are most likely to benefit from therapeutic intervention is vital. The aim of this study was to evaluate the role and safety of cardiac MR (CMR) in the investigation of patients successfully resuscitated from out of hospital sudden cardiac arrest.

## Methods

A retrospective study was performed of all patients referred to our institution between July 2006 and July 2011 for CMR evaluation following successfully resuscitated out of hospital sudden cardiac arrest. Patients were defined as having a likely coronary disease aetiology vs non-coronary disease aetiology based on cardiac risk factors, echocardiography, post-resuscitation and admission ECG findings prior to CMR. CMR imaging was blindly interpreted to assign each patient to either a coronary disease aetiology or non-coronary disease aetiology groups based on CMR evaluation. The results were subsequently compared with the post event coronary angiogram.

## Results

A total of 79 patients underwent CMR evaluation following successfully resuscitated cardiac arrest. In total 55.7% of patients had a coronary disease aetiology and 44.3% had a non-coronary disease aetiology. CMR changed the diagnosis in 20% of patients with a pre-CMR likely diagnosis of coronary disease and 13.3% of patients with a pre-CMR likely diagnosis of non-coronary disease. All our patients tolerated the entire CMR study with no adverse events.

## Conclusions

CMR is a safe technique in the investigation of patients successfully resuscitated following out of hospital sudden cardiac arrest and identifies a non-coronary cause in a significant number of cases.

## Funding

No external funding.

